# Double‐Network DNA Macroporous Hydrogel Enables Aptamer‐Directed Cell Recruitment to Accelerate Bone Healing

**DOI:** 10.1002/advs.202303637

**Published:** 2023-11-10

**Authors:** Yali Miao, Xiao Liu, Jinshui Luo, Qian Yang, Yunhua Chen, Yingjun Wang

**Affiliations:** ^1^ School of Materials Science and Engineering South China University of Technology Guangzhou 510641 China; ^2^ National Engineering Research Center for Tissue Restoration and Reconstruction South China University of Technology Guangzhou 510006 China; ^3^ Department of Orthopedics Guangdong Provincial People's Hospital Guangdong Academy of Medical Sciences Guangzhou 510080 China; ^4^ Guangdong Cardiovascular Institute Guangdong Provincial People's Hospital Guangdong Academy of Medical Sciences Guangzhou 510080 China; ^5^ Key Laboratory of Biomedical Engineering of Guangdong Province and Innovation Center for Tissue Restoration and Reconstruction South China University of Technology Guangzhou 510006 China; ^6^ Key Laboratory of Biomedical Materials and Engineering of the Ministry of Education South China University of Technology Guangzhou 510006 China

**Keywords:** air‐in‐water emulsion, bone regeneration, cell recruitment, DNA hydrogels, double network

## Abstract

Recruiting endogenous bone marrow mesenchymal stem cells (BMSCs) in vivo to bone defect sites shows great promise in cell therapies for bone tissue engineering, which tackles the shortcomings of delivering exogenous stem cells, including limited sources, low retention, stemness loss, and immunogenicity. However, it remains challenging to efficiently recruit stem cells while simultaneously directing cell differentiation in the dynamic microenvironment and promoting neo‐regenerated tissue ingrowth to achieve augmented bone regeneration. Herein, a synthetic macroporous double‐network hydrogel presenting nucleic acid aptamer and nano‐inducer enhances BMSCs recruitment, and osteogenic differentiation is demonstrated. An air‐in‐water template enables the rapid construction of highly interconnective macroporous structures, and the physical self‐assembly of DNA strands and chemical cross‐linking of gelatin chains synergistically generate a resilient double network. The aptamer Apt19S and black phosphorus nanosheets‐specific macroporous hydrogel demonstrate highly efficient endogenous BMSCs recruitment, cell differentiation, and extracellular matrix mineralization. Notably, the enhanced calvarial bone healing with promising matrix mineralization and new bone formation is accompanied by adapting this engineered hydrogel to the bone defects. The findings suggest an appealing material approach overcoming the traditional limitations of cell‐delivery therapy that can inspire the future design of next‐generation hydrogel for enhanced bone tissue regeneration.

## Graphical abstract

A double‐network DNA macroporous hydrogel imparted with nucleic acid aptamer and nano‐inducer is developed to enhance BMSCs recruitment and osteogenic differentiation. The air‐in‐water template enables the rapid construction of highly interconnective macroporous structures. The resilient macroporous composite hydrogel with dual bioagents efficiently promotes endogenous BMSCs recruitment, cell differentiation, extracellular matrix mineralization, and achieves enhanced in vivo calvarial bone healing.

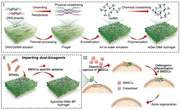

## 1 Introduction

As a highly mineralized connective tissue, bone injuries can lead to severe pain and disability, and bone tissue reconstruction is still facing considerable challenges. Tissue engineering method of integrating cells, signaling molecules, and/or biological materials to repair damaged tissues has provided great potentials.^[^ ^1^ ^]^ Utilizing mesenchymal stem cells (MSCs) to transplant and further stimulate their differentiation into osteoblasts through the assistance of implanted materials has been preferably adopted to accelerate bone tissue repair.^[^ ^2^ ^]^ Imparting bioactive nano agents into implant materials can effectively direct cell behavior.^[^ ^3^ ^]^ Black phosphorus nanosheets (BPNSs) with a thermodynamically stable phosphorus allotrope can provide the necessary phosphorus to promote stem cell osteogenesis, which has been utilized for enhanced bone regeneration and repair.^[^ ^4^ ^]^ Phosphorus‐rich implant materials can participate in the regulation of the bone tissue microenvironment by increasing the local phosphate concentration to provide nucleation sites for calcium deposition and matrix mineralization to guide bone regeneration.^[^ ^5^ ^]^ Specifically, the degradation of BPNSs occurs preferentially in the phosphorus atoms at the edge of BPNSs, and the polyphosphate radicals formed by edge oxidation are analogous to bisphosphonic acid, a drug used to treat osteoporosis. The polyphosphate radicals generated by the oxidative degradation of BPNSs can attract free calcium ions to form calcium a phosphate mineralized matrix, thus promoting bone regeneration.^[^ ^6^ ^]^

However, exogenous stem cell transplantation faces its inevitable shortcomings in bone tissue engineering, including the lack of a MSCs‐targeted delivery system, the low survival rate of exogenous cells, and the risk of exogenous infection caused by in vitro cell culture.^[^ ^7^ ^]^ The emergence of regeneration methods based on endogenous MSCs recruitment provides a new perspective for stem cell‐based bone tissue engineering.^[^ ^8^ ^]^ It is increasingly recognized that mediating endogenous MSCs homing preferably promotes bone defect repair.^[^ ^9^ ^]^ The recruitment of endogenous MSCs can avoid the above shortcomings of in vitro delivery of stem cells.^[^ ^10^ ^]^ However, to achieve high‐efficiency of MSCs recruitment, it is important to select the appropriate chemotactic agents. Aptamers, especially ssDNA or RNA oligonucleotides, are interesting candidates that have been used for disease diagnosis, treatment, and tissue engineering.^[^ ^11^ ^]^ Compared with antibodies and peptides, nucleic acid aptamers demonstrate obvious advantages, including small physical size, tailorable structure, facile chemical modification, low immunogenicity, and high stability.^[^ ^12^ ^]^ Recently, the Apt19S nucleic acid aptamer was found to specifically bind to stem cells with high affinity,^[^ ^13^ ^]^ which has attracted widespread attention in stem cell recruitment.^[^ ^14^ ^]^ Regarding cell behavior and the surrounding microenvironment, another question that arises is stem cell osteogenesis after the recruitment of bone defects. This requires the implanted materials to have the ability of regulating cell differentiation.

From the structural view of implant materials, it is also important to build interconnective macropores inside the materials to support cell infiltration and bone tissue ingrowth.^[^ ^15^ ^]^ Taking hydrogel‐based implant materials as examples, they are composed of biocompatible natural and/or synthetic polymers with low immunogenicity, performance adjustability, biodegradability, and processability,^[^ ^16^ ^]^ which are widely developed for bone regenerative applications.^[^ ^17^ ^]^ However, the dense structure of hydrogels limits cell migration and new bone ingrowth. Compared to dense hydrogels, macroporous hydrogel scaffolds suffer from relatively weak mechanical strength due to the loose porous structures. Notably, by introducing a second cross‐linking network into macroporous hydrogels, the two intertwined cross‐linked networks can integrate the physicochemical properties of two different networks, thereby improving or imparting specific properties, including mechanical strength.^[^ ^18^ ^]^ Moreover, the additional polymer could improve the emulsion stability through the viscosity adjustment of the aqueous continuous phase.^[^ ^19^ ^]^ Interestingly, DNA, the core genetic material that has complementary pairing bases, enables precise and efficient self‐assembly capabilities to form a physical cross‐linking network in water.^[^ ^20^ ^]^ DNA is biocompatible and biodegradable, and the sequences are highly adjustable.^[^ ^21^ ^]^ The reversible and relatively strong network endows an energy dissipation mechanism, providing the possibility to develop resilient interpenetrating double‐network matrices.^[^ ^22^ ^]^ Therefore, a DNA‐reinforced hydrogel matrix with macroporous structures and sufficient mechanical strength that can recruit stem cells, direct differentiation, and simultaneously promote cell migration and tissue growth offers a unique opportunity for enhancing bone tissue regeneration, but the relevant studies remain unexplored.

**Figure 1:**
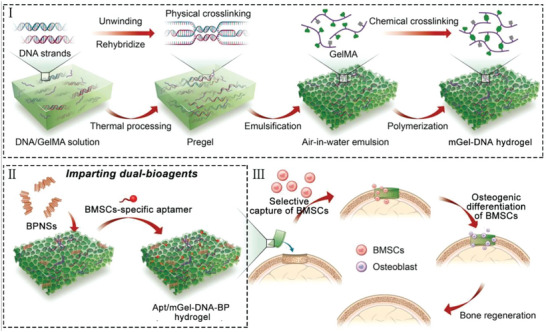
Schematic illustration of designing mGel‐DNA double‐network macroporous hydrogels with imparting dual‐bioagents Apt19S and BPNSs to recruit and differentiate stem cells for augmented bone regeneration.

Herein, we develop a double‐network macroporous hydrogel (mGel‐DNA) conveniently constructed by the air‐in‐water emulsion template for promoting bone regeneration (**Figure** **1**). The double physical/chemical cross‐linking network composed of DNA and gelatin, designed with a highly interconnective macroporous structure, aims to maintain good mechanical strength while simultaneously promoting cell infiltration and tissue ingrowth. Apt19S is selected and chemically anchored the hydrogel network to recruit the endogenous BMSCs, while the imparted BPNSs regulate osteogenesis and coordinate with calcium ions to form calcium phosphate for mineralization. Utilizing this mGel‐DNA hydrogel as a model, we explore the synergistic effect of porous structure, bioactive nanoagent, and recruited endogenous stem cells in promoting bone repair and proposed a potentially interesting method to develop functional bio‐scaffolds for augmenting bone tissue regeneration.

## 2 Results and Discussion

### 2.1 Preparation of Double‐Network Macroporous mGel‐DNA Hydrogel

**Figure 2:**
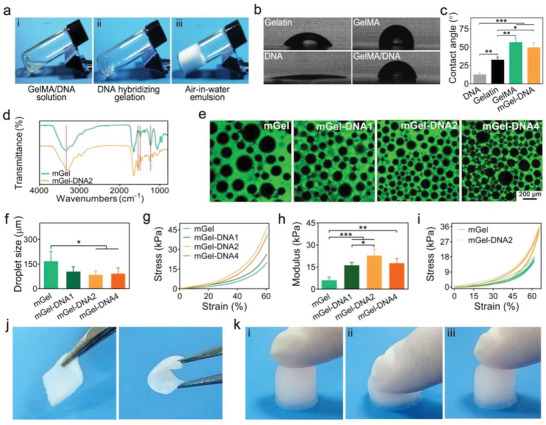
a) Stable air‐in‐water emulsions formed by mechanical shearing the GelMA/DNA solution, (i) GelMA/DNA solution, (ii) GelMA/DNA hybridizing gelation induced by heating and cooling, (iii) the formed emulsions showing gelling state. b) Water droplets and c) contact angles on various bulky hydrogels (Gelatin, GelMA, DNA, GelMA/DNA). d) FTIR spectra of mGel and mGel‐DNA hydrogels. e) Fluorescence images of air‐in‐water emulsion droplets stabilized by GelMA and DNA with various concentrations. f) Droplet size of mGel‐DNA emulsions. g–i) Mechanical properties of the macroporous mGel hydrogel and mGel‐DNA double‐network macroporous hydrogels: (g) Stress–strain curves, (h) compressive modulus, i) cyclic compression curves. j) Photographs of the mGel‐DNA macroporous hydrogel before and after bending with tweezers, and k) photographs of the resilient mGel‐DNA macroporous hydrogel pressed by finger. ^*^ *p* < 0.05, ^**^ *p* < 0.01, ^***^ *p* < 0.001.

Gelatin is a hydrophilic natural polymer with limited emulsification ability, however, after modification with methacryloyl moieties, the prepared GelMA polymer turns into a good surfactant to stabilize emulsions. It is known that stable emulsions can serve as promising templates for the preparation of macroporous bioscaffolds. In particular, air‐in‐water emulsions without using organic solvents show great benefits since the biocompatibility issues of possible solvent residues in the scaffolds are eliminated. Herein, GelMA with an 80% methacryloyl substituent ratio was applied to form many air‐in‐water emulsion droplets through high‐speed mechanical stirring, providing a highly stable emulsion template for the preparation of macroporous functional hydrogels (**Figure** **2**a). The wettability of gelatin before and after modification was characterized by the contact angle test (Figure 2b,c). The test was conducted by applying water droplets on various bulky hydrogels with compositions of gelatin, GelMA, DNA, and GelMA/DNA, respectively. The average contact angle of gelatin before modification was 32.9°, showing a relatively hydrophilic state, while the average contact angle of GelMA was increased to 56.7° after modification. The methylacryloyl moieties were grafted onto gelatin chains via reaction with the amino groups, leading to an increase in the amphiphilicity of GelMA. Notably, the introduction of DNA strands into the aqueous phase greatly enhanced the emulsification efficiency and emulsion stability (Figures S1 and S2, Supporting Information). This might be attributed to the increased interactions of mGel‐DNA2 emulsion droplets compared to pristine GelMA emulsions (Figure S3, Supporting Information). Furthermore, with the introduction of DNA, the zeta potential of GelMA significantly increased, as shown in Figure S4 (Supporting Information). The electrostatic attraction between the electropositive region of the GelMA molecular chain with the negatively charged oxygen of the phosphate group destabilizes the electronic environment of the nearest phosphorus atom. This leads to a decrease in electron density around phosphorus, resulting in a significant change in the Zeta potential of the GelMA/DNA mixture compared to GelMA and DNA. The above results all indicated that a significant mutual attraction between GelMA molecular chains and the DNA skeleton.

Air‐in‐water GelMA emulsions with different DNA concentrations (0, 1, 2, and 4 w/v%) were constructed and labeled as mGel, mGel‐DNA1, mGel‐DNA2, and mGel‐DNA4, respectively. After leaving the emulsion at room temperature for 12 h, there was no obvious demulsification or delamination in the mGel‐DNA composite emulsions compared with the pristine mGel emulsion, indicating that the existence of the DNA physical network can improve the stability of the mGel emulsion (Figure S2, Supporting Information). To further identify the interactions between GelMA and the DNA molecular chains, FTIR characterization was carried out on the hydrogels with and without DNA (Figure 2d). The mGel hydrogel showed the amino characteristic peak of gelatin near 1540 cm^−1^. After the introduction of DNA, due to the hydrogen bond interaction between GelMA and DNA strands, the amino characteristic peak at 1540 cm^−1^ became significantly wider. In addition, the characteristic peaks at 1235, 1493, and 3330 cm^−1^ also shifted in mGel‐DNA hydrogel. These findings reveal the interactions between GelMA and DNA.

The air bubbles in the emulsions can be clearly observed by fluorescence microscopy, as GelMA is tagged with fluorescein isothiocyanate (FITC) (Figure 2e). With the addition of DNA into the aqueous solution, the droplet size of mGel‐DNA composite emulsions was significantly reduced and distributed between 50 and 150 µm (Figure 2f). We estimated that the DNA physical cross‐linking network can increase the viscosity of the continuous phase of the gel emulsion and delay the migration rate of the emulsion droplets, thereby preventing the droplets from coalescing in the emulsion and forming a more stable emulsion template.

The emulsions were solidified to produce composite macroporous hydrogels through UV photoinitiated radical polymerization of the continuous phase. Dynamic thermomechanical analysis (DMA) was first used to characterize the mechanical properties of the resulting hydrogels, and the stress–strain curves are shown in Figure 2g. The compression moduli of the mGel‐DNA macroporous hydrogels were significantly higher than that of the mGel macroporous hydrogel (6 kPa), while the mGel‐DNA2 macroporous hydrogel exhibited the highest moduli with a value of 22 kPa (Figure 2h). The mGel‐DNA4 hydrogel showed a decreased mechanical modulus, which could be possibly attributed to the inhomogeneous emulsification and larger emulsion droplet size compared to the mGel‐DNA2 macroporous hydrogel, thus resulting in the non‐uniformity of stress distribution of macroporous hydrogel. The mechanical reinforcement of DNA in the macroporous hydrogels was also confirmed by rheological characterization (Figure S5, Supporting Information). DNA self‐assembly induced the formation of a physical network, which promoted a significant increase in the storage modulus (G’) of mGel‐DNA macroporous hydrogels compared to the mGel macroporous hydrogel.

Moreover, both mGel and mGel‐DNA2 macroporous hydrogels showed good shape recovery under cyclic compression tests (Figure 2i), and the mGel‐DNA2 macroporous hydrogel can be bent without deformation (Figure 2j). When axial pressure was applied to the mGel‐DNA2 macroporous hydrogel with a finger, the hydrogel had no obvious damage, and after the pressure was removed, it quickly returned to its original state (Figure 2k). The synergistic effect of this double‐network enables the hydrogel to be superior to the single‐network hydrogel in mechanical strength.^[^ ^23^ ^]^ The chemically cross‐linked GelMA network is rigid, while the physical network of DNA is relatively soft but reversible. The two networks worked together to achieve effective energy dissipation and enhanc the mechanical strength of the macroporous hydrogel.

### 2.2 Interconnective Macroporous Structures of mGel‐DNA Hydrogels

**Figure 3:**
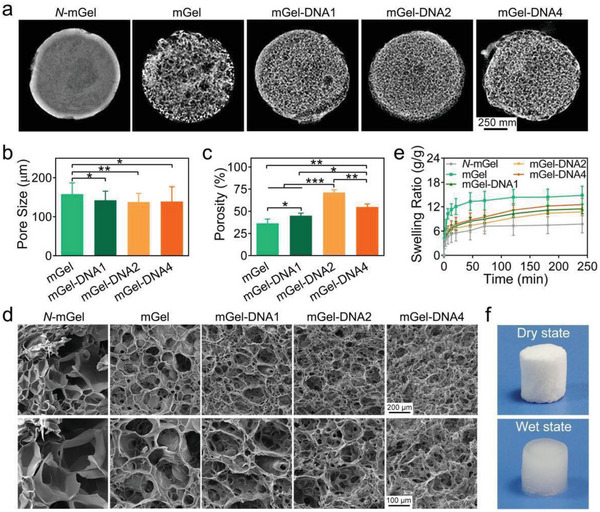
a) Micro‐CT images of the *N*‐mGel nonporous hydrogel, mGel, and mGel‐DNA macroporous hydrogels. b) Pore size and c) porosity of mGel and mGel‐DNA macroporous hydrogels. d) SEM images of mGel‐DNA hydrogels showing interconnective macroporous structures. e) Swelling curves of lyophilized *N*‐mGel nonporous hydrogel, mGel, and mGel‐DNA macroporous hydrogels. f) Photos of the freeze‐dried mGel‐DNA macroporous hydrogel before and after absorbing water. ^*^ *p* < 0.05, ^**^ *p* < 0.01, ^***^ *p* < 0.001.

Imparting macroporous structure into hydrogels can promote the ingrowth of bone tissue, which is regarded as an important feature to enhance the bone repair performance of implants.^[^ ^24^ ^]^ The mGel macroporous hydrogel presented an opaque white appearance, while the dense nonporous hydrogel was slightly transparent (Figure S6, Supporting Information). The macroporous structures of mGel‐DNA hydrogel were investigated by 3D reconstruction topography and scanning electronic microscopy (**Figure** **3**a–d).

The dense *N*‐mGel hydrogel formed a porous structure without a pore window structure after the ice crystals were sublimated. In contrast, mGel and mGel‐DNA macroporous hydrogels prepared by the air‐in‐water emulsion templates showed highly interconnective macroporous structures with clear pore window structures. The mGel‐DNA2 macroporous hydrogel had the best pore uniformity and improved pore connectivity. The porosity of the mGel‐DNA2 macroporous hydrogel was 71.3 ± 3.1%, and the pore size distribution was ≈137 µm. Notably, when the DNA concentration increased to 4 w/v%, partially closed pores appeared inside the hydrogel. This phenomenon can be attributed to the high concentration of DNA making the emulsion system too viscous, resulting in an inhomogeneous distribution of emulsion droplets, thereby decreasing the pore connectivity.

mGel‐DNA emulsions showed promising injectability. For instance, the mGel‐DNA2 emulsion containing the photoinitiator was injected into the PBS solution through a syringe needle at room temperature, and the emulsion can be stably immersed in PBS without obvious collapse, and an emulsion gel filament was formed after UV‐induced free radical polymerization (Figure S7, Supporting Information). In addition, we further evaluated the degradation of the macroporous hydrogels in the presence of collagenase Type II (Figure S8, Supporting Information). It clearly showed that the degradation of hydrogels is retardant with the incorporation of DNA, which was probably due to the condenser cross‐linking network of hydrogels with more imparted DNA macromolecules. Furthermore, from the swelling curves of the macroporous hydrogels in Figure 3e, it can be found that mGel‐DNA macroporous hydrogels exhibited a lower swelling rate than mGel macroporous hydrogels due to the physical/chemical double cross‐linking network of hydrogels. As shown in Figure 3f, we found that the porous interior of mGel‐DNA2 macroporous hydrogel was filled with water after long‐term immersion, and its original shape did not change significantly, which was due to the formation of a relatively dense double cross‐linking network of mGel‐DNA2 macroporous hydrogel, thus inhibiting the morphological change of the macroporous hydrogel. Therefore, mGel‐DNA2 macroporous hydrogel can be used as bone repair implants without compressing the surrounding soft tissues and nerves near the bone.

### 2.3 Proliferation and Differentiation of BMSCs in mGel‐DNA‐BP Hydrogels

The above physical and chemical characterization results clearly showed that DNA participates in the formation of a stable GelMA emulsion, particularly at a concentration of 2 w/v%, allowing the construction of a homogeneous mechanically enhanced macroporous hydrogel matrix. Therefore, the mGel‐DNA2 macroporous hydrogel was selected for subsequent in vitro biological research. Furthermore, BPNSs were used as an osteogenic active component to be incorporated into the mGel‐DNA2 double cross‐linked network, and the specific contents of BPNSs in mGel‐DNA2 hydrogels are shown in Table S1, Supporting Information. The BPNSs used in our study were prepared by the liquid phase stripping method, and the morphology is shown in Figure S9 (Supporting Information). The introduction of BPNSs slightly enhanced the mechanical strength of macroporous hydrogels, and the mechanical flexibility was maintained (Figures S10 and S11, Supporting Information).

**Figure 4:**
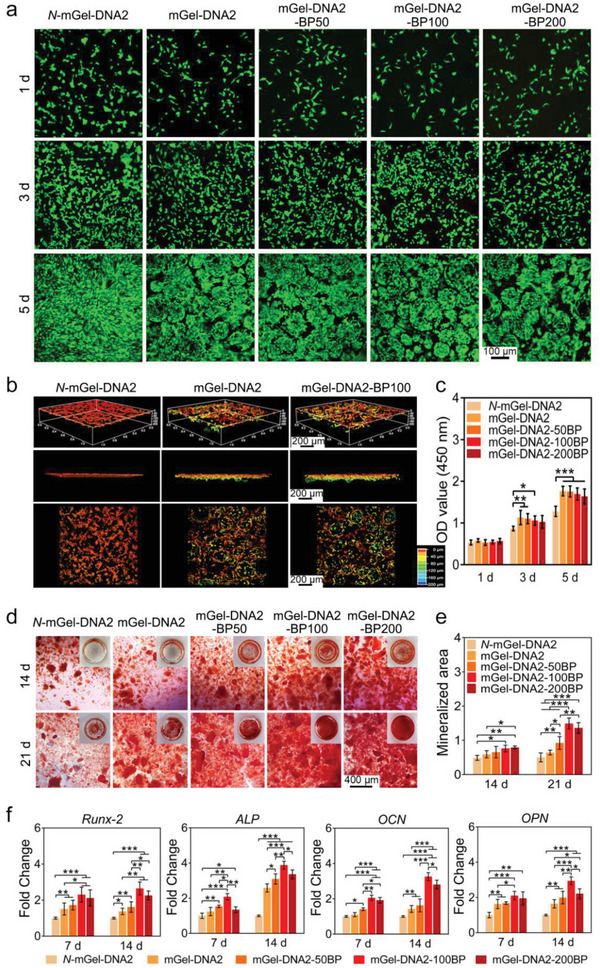
a) Live‐dead fluorescence images of BMSCs cultured on hydrogels for 1, 3, and 5 days. b) CLSM images of BMSCs distributed in macroporous hydrogels. c) CCK‐8 test of BMSCs cultured on hydrogels for 1, 3, and 5 days. d,e) The mineralized matrix of BMSCs cultured on mGel‐DNA2 and mGel‐DNA2‐BP macroporous hydrogels for 14 and 21 days. f) Expression of osteogenic genes of BMSCs cultured on mGel‐DNA2 and mGel‐DNA2‐BP macroporous hydrogels for 7 and 14 days, including *Runx‐2*, *ALP*, *OCN* and *OPN*. ^*^ *p* < 0.05, ^**^ *p* < 0.01, ^***^ *p* < 0.001.

Rat bone marrow mesenchymal stem cells (BMSCs) were used to evaluate the effect of hydrogel on cell viability. The live‐dead staining results showed that BMSCs maintained good proliferation behavior on the hydrogels without obvious cytotoxicity, and BMSCs adhered to the inside and edges of the porous structure in the hydrogels (**Figure** **4**a). Interestingly, BMSCs clusters/spheres were formed in the mGel‐DNA2 and mGel‐DNA2‐BP macroporous hydrogels, which could be attributed to the curvature and GelMA distributed at the macropore surface, effectively promoting the adhesion and 3D growth of BMSCs inside the macropores. After BMSCs were cultured on nonporous and porous hydrogels for 3 days, BMSCs were labeled with calcein‐AM staining, and the fluorescence images in the 3D mode are shown in Figure 4b. BMSCs in different depth ranges were distinguished by different colors, and BMSCs distribution in hydrogels was analyzed. BMSCs seeded on the *N*‐mGel‐DNA2 nonporous hydrogel cannot penetrate into the hydrogel after 3 days of culture; in contrast, BMSCs seeded on the mGel‐DNA2 and mGel‐DNA2‐BP macroporous hydrogels can migrate to the porous interior of the hydrogels and adhere along the pore surface. The penetration depth of BMSCs in both mGel‐DNA2 and mGel‐DNA2‐BP100 macroporous hydrogels can reach 120 µm (Figure S12, Supporting Information). Notably, the porous structure inside the hydrogels was interlaced, and the depth of confocal observation was limited, so it was difficult to observe cells deeper inside the hydrogels. Moreover, from the CCK‐8 assay result in Figure 4c, it can be seen that the viability of BMSCs on the macroporous hydrogels was significantly higher than that on the nonporous hydrogel as the culture time increased, indicating that the double‐network macroporous hydrogels with BPNSs showed good cell compatibility and that the 3D space of the macroporous hydrogels provided more sufficient space for BMSCs proliferation.

Alizarin red staining was used to analyze the formation of mineralized matrix, and the red area was the mineralized matrix formed by BMSCs (Figure 4d). On days 14 and 21, the *N*‐mGel‐DNA2 nonporous hydrogel had only sporadic red areas, indicating that only a small amount of mineralized matrix was formed by BMSCs. In contrast, mGel‐DNA2‐BP macroporous hydrogels had significantly more mineralized matrix formation without osteogenic factors, which was consistent with the quantitative analysis results (Figure 4e). To assess the effect of mGel‐DNA2‐BP macroporous hydrogels on the expression of osteogenic genes, key osteogenic genes (*Runx‐2*, *ALP*, *OCN*, and *OPN*) were further evaluated by a quantitative qRT‐PCR assay (Figure 4f). In the early stage of osteogenesis (day 7), mGel‐DNA2‐BP100 and mGel‐DNA2‐BP200 macroporous hydrogels significantly upregulated the expression of osteogenic genes. In particular, the expression of Runx‐2 in the mGel‐DNA2‐BP100 macroporous hydrogel was 2.6 ± 0.4 times that in the *N*‐mGel‐DNA2 nonporous hydrogel. It was worth noting that the mGel‐DNA2 macroporous hydrogel enhanced the expression of Runx‐2 and OPN compared with the *N*‐mGel‐DNA2 nonporous hydrogel. Werner et al. reported that the microenvironmental signals of implanted materials affect cell behavior, and the curvature of the 3D substrate can change cell adhesion morphology, thereby affecting the cytoskeleton force acting on the nucleus and mediating the osteogenic differentiation of cells.^[^ ^25^ ^]^ The porous structure resulted in the different morphologies of cell adhesion between the *N*‐mGel‐DNA2 nonporous hydrogel and the mGel‐DNA2 hydrogel, such as cell orientation, which was also one of the possible reasons for the difference in cell gene expression between porous hydrogels and nonporous hydrogels. In the late stage of osteogenic differentiation (day 14), the expression levels of these four osteogenic‐related genes remained at a high level in the mGel‐DNA2‐BP100 and mGel‐DNA2‐BP200 macroporous hydrogel groups.

The above experiments revealed that the double‐network mGel‐DNA2 macroporous hydrogel can provide a 3D matrix environment for the osteogenic differentiation of BMSCs. Overall, it demonstrated that BPNSs effectively stimulated the directional osteogenic differentiation of BMSCs, thereby enhancing the osteoinductive activity of mGel‐DNA2 macroporous hydrogel.

### 2.4 Apt19S‐Pendant mGel‐DNA‐BP Hydrogel to Bind BMSCs

**Figure 5:**
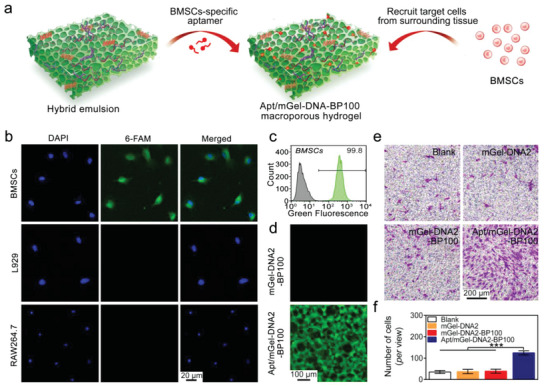
BMSCs recognition and binding ability of the Apt/mGel‐DNA2‐BP100 hydrogel. a) Schematic diagram of the preparation of Apt19S‐functionalized mGel‐DNA2‐BP100 macroporous hydrogel and cell binding. b) Fluorescence images of BMSCs, L929 cells, and RAW264.7 cells after co‐incubation with 6‐FAM‐labeled Apt19S, respectively, indicating that Apt19S can specifically bind to BMSCs. c) Flow cytometry of BMSCs incubated with 6‐FAM‐labeled Apt19S. The green peak represents Apt19S‐bound BMSCs. d) Fluorescence images of mGel‐DNA2‐BP100 and Apt/mGel‐DNA2‐BP100 macroporous hydrogels, the green fluorescence representing 6‐FAM‐labeled Apt19S. e,f) Chemotactic behavior of BMSCs on mGel‐DNA2, mGel‐DNA2‐BP100, and Apt/mGel‐DNA2‐BP100 macroporous hydrogels showing that the Apt/mGel‐DNA2‐BP100 macroporous hydrogel can effectively recruit more BMSCs. ^*^ *p* < 0.05, ^**^ *p* < 0.01, ^***^ *p* < 0.001.

Recruiting endogenous stem cells to bone defect sites and promoting osteogenic differentiation can significantly enhance tissue regeneration.^[^ ^12^, ^26^ ^]^ A BMSCs‐specific aptamer Apt19S with high efficiency and stability was selected and chemically functionalized with acrydite at its 5′‐end for anchoring to the mGel‐DNA2‐BP100 macroporous hydrogel to achieve BMSCs recruitment (**Figure** **5**a). We first verified the specific binding ability of Apt19S to stem cells. The 3′end of Apt19S labeled with 6‐FAM was incubated with BMSCs, L929, and RAW264.7 cells. There was almost no green fluorescence detected on the cell membrane of L929 and RAW264.7 cells, while BMSCs clearly showed fluorescence labeling of 6‐FAM, indicating that Apt19S can selectively bind to BMSCs (Figure 5b). Additionally, after the co‐incubation of BMSCs with 6‐FAM‐labeled Apt19S, the quantitative fluorescence intensity of BMSCs was significantly enhanced, and the proportion of BMSCs with bound Apt19S was as high as 99.8%, confirming the specific binding ability of Apt19S to BMSCs (Figure 5c).

To observe the distribution of Apt19S on the surface of the mGel‐DNA2‐BP100 macroporous hydrogel, we used 6‐FAM fluorescent moieties to label Apt19S. As shown in Figure 5d, there were obvious fluorescence signals in the Apt/mGel‐DNA2‐BP100 macroporous hydrogel sample, indicating that Apt was successfully incorporated into the hydrogel network. Notably, Apt19S exerted no effect on the macroscopic appearance and mechanical properties of the macroporous hydrogel. A transwell experiment was performed to assay the chemotactic effect of macroporous hydrogels on BMSCs. The Apt/mGel‐DNA2‐BP100 macroporous hydrogel recruited more BMSCs to the lower surface of the upper chamber than the other groups (Figure 5e,f), which proved that the Apt/mGel‐DNA2‐BP100 macroporous hydrogel effectively retained the high specific affinity of Apt19S for stem cells.

### 2.5 In Vivo BMSCs Recruitment with Apt19S‐Pendant mGel‐DNA‐BP Hydrogel

**Figure 6:**
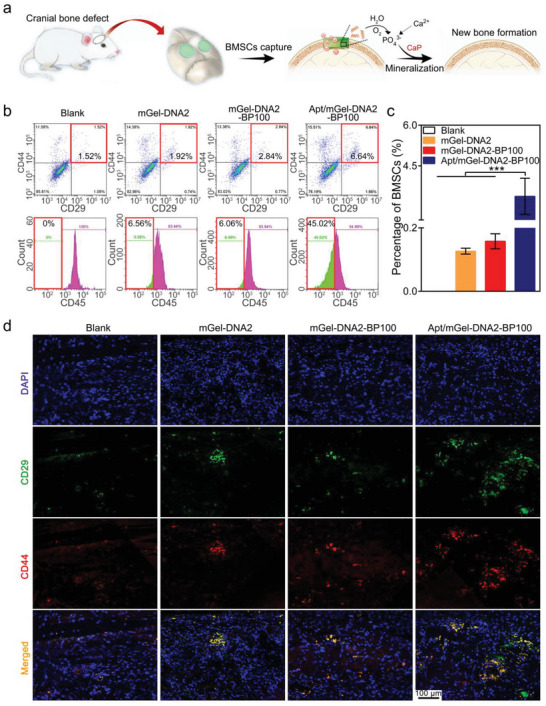
In vivo BMSCs recruitment. a) Schematic diagram of recruitment and differentiation of stem cells induced by Apt/mGel‐DNA2‐BP100 macroporous hydrogel in cranial bone defect. b) Classification gate flow cytometric analysis of Apt/mGel‐DNA2‐BP100 macroporous hydrogel to recruit endogenous BMSCs in cranial defect. Cells with double‐positive expression of CD29 and CD44 (top row) and CD45‐negative cells (bottom row) were regarded as BMSCs. c) Quantitative analysis of the percentage of endogenous BMSCs recruited by the Apt/mGel‐DNA2‐BP100 macroporous hydrogels in cranial defects. d) Immunofluorescence staining images of CD29‐positive and CD44‐positive BMSCs recruited by the Apt/mGel‐DNA2‐BP100 macroporous hydrogel in cranial defects. ^*^ *p* < 0.05, ^**^ *p* < 0.01, ^***^ *p* < 0.001.

We constructed a Sprague‐Dawley rat cranial defect to evaluate the in vivo stem cell recruitment and bone repair effects of the Apt19S‐functionalized macroporous hydrogels (**Figure** **6**a). The operation process is shown in Figure S13 (Supporting Information). We conducted flow cytometry to identify specific antigens (CD29, CD44, and CD45). CD45‐negative cells were selected from CD29 and CD44 double‐positive cells, and the percentage of these cells in the total number of cells was calculated, which was regarded as the percentage of BMSCs in the bone defect. As shown in Figure 6b,c, the cells with double‐positive expression of CD29 and CD44 in the Apt/mGel‐DNA2‐BP100 macroporous hydrogel group accounted for 6.6%, which was significantly higher than that in the other groups. Among these double‐positive cells, the proportion of cells with negative expression of CD45 was as high as 45.0%, indicating that the number of BMSCs recruited by the Apt/mGel‐DNA2‐BP100 macroporous hydrogel group was significantly greater than that of the other groups.

Immunofluorescence of tissue sections was used to analyze the cell types in the bone defect area, and CD29 and CD44 were selected as the specific antigens of stem cells. The CD29 label was green, the CD44 label was red, and the blue fluorescent dot was the cell nucleus. As shown in Figure 6d, the green and red fluorescent dots in the Apt/mGel‐DNA2‐BP100 macroporous hydrogel were significantly more abundant than those in the mGel‐DNA2 and mGel‐DNA2‐BP100 macroporous hydrogels, and the overlapping area appeared as bright yellow cell dots. Double‐positive expression of CD29 and CD44 in the Apt/mGel‐DNA2‐BP100 macroporous hydrogel was significantly higher than that in the other groups. There was obvious BMSCs enrichment in the bone defect. Therefore, we concluded that the Apt/mGel‐DNA2‐BP100 macroporous hydrogel can effectively drive the migration of endogenous BMSCs to the bone defect site and provide a favorable cellular environment for efficiently exerting the osteoinductive activity of BPNSs.

### 2.6 In Vivo Bone Regeneration with Apt19S‐Pendant mGel‐DNA‐BP Hydrogel

**Figure 7:**
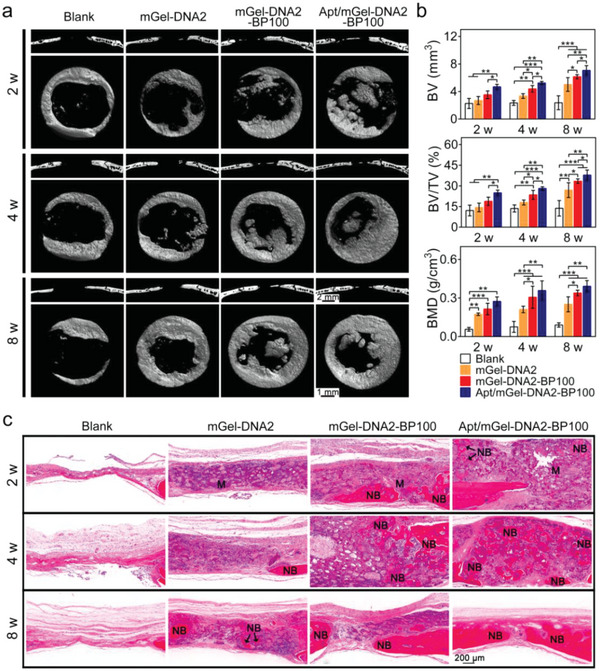
Augmenting bone regeneration by Apt/mGel‐DNA2‐BP100 macroporous hydrogel. a) Representative micro‐CT image of bone defects treated with mGel, mGel‐DNA2‐BP100, and Apt/mGel‐DNA2‐BP100 macroporous hydrogels post‐implantation for 2, 4, and 8 weeks. b) Quantitative analysis of osteogenesis‐related parameters based on the 3D reconstruction micro‐CT images of different groups. c) H&E staining images of the regenerated bone, *NB* stands for new bone, *M* stands for implanted hydrogel materials.

The bone repair performance of macroporous hydrogels was also evaluated by the cranial defect model. Sprague‐Dawley rats were sacrificed, and tomographic analysis of cranial defects was performed by micro‐CT (**Figure** **7**a). At three different time points, the blank group presented large bone loss without obvious new bone formation. At the 2nd and 4th weeks, the new bone at the defect site of macroporous hydrogels mainly grew from the edge to the center of the defects. At the 8th week, the thickness of new bone in the mGel‐DNA2‐BP100 and Apt/mGel‐DNA2‐BP100 macroporous hydrogels obviously increased and was significantly higher than that in the blank group and mGel‐DNA2 macroporous hydrogel.

Consistent with the micro‐CT qualitative results, the implantation of macroporous hydrogels containing BPNSs significantly increased the relevant parameters of new bone in cranial defects (Figure 7b), including new bone volume (BV), new bone volume/defect volume (BV/TV), and new bone density (BMD). At week 8, the BV of the mGel‐DNA2‐BP100 macroporous hydrogel group (6.14 ± 0.31 mm^3^) was higher than that of the blank group (2.37 ± 1.01 mm^3^) and the mGel‐DNA2 macroporous hydrogel group (5.02 ± 1.00 mm^3^), and the BV of the Apt/mGel‐DNA2‐BP100 macroporous hydrogel group (7.07 ± 0.69 mm^3^) was the highest. Similarly, the BMD of the mGel‐DNA2 macroporous hydrogel group (0.25 ± 0.06 g cm^−3^), mGel‐DNA2‐BP100 macroporous hydrogel group (0.34 ± 0.02 g cm^−3^), and Apt/mGel‐DNA2‐BP100 macroporous hydrogel group (0.39 ± 0.04 g cm^−3^) was also significantly higher than that of the blank group (0.09 ± 0.02 g cm^−3^). It can be concluded that mGel‐DNA2‐BP100 and Apt/mGel‐DNA2‐BP100 macroporous hydrogels can promote the regeneration of bone tissue, and the enrichment of stem cells in the bone defect area further enhanced the upregulation effect of mGel‐DNA2‐BP100 macroporous hydrogel on new bone formation.

The H&E staining images in Figure 7c clearly showed that there was a large amount of fibrous tissue in the bone defect area without material implanted. As the post‐implantation time was prolonged, there was much newly formed bone in the porous interior of the Apt/mGel‐DNA2‐BP100 macroporous hydrogel, indicating that BPNSs and Apt19S not only synergistically improved the osseointegration of the hydrogel but also provided a suitable matrix microenvironment for bone ingrowth. Masson staining can be conducted to analyze the formation of collagen fibers in the bone defect area and to evaluate the formation and maturity of bone tissue. As shown in Figure S14 (Supporting Information), the implanted hydrogels presented an obvious porous structure at the 2nd week, providing a suitable 3D environment for cell migration and tissue growth. The cranial defect of the blank group was filled with a large amount of loose connective tissue, and there was no obvious formation of collagen fibers. At the 4th week, the new bone matrix can be embedded in the mGel‐DNA2‐BP100 macroporous hydrogel to form a mixed layer of material and bone, and this mixed layer can improve the mechanical interlocking between the implant and the host bone, which is the key interface for providing mechanical stability. Meanwhile, Apt/mGel‐DNA2‐BP100 rapidly recruited more BMSCs to participate in bone formation and further promoted a large number of discontinuous new bones filling the porous interior in a form similar to bone islands. At the 8th week, the porous hydrogel gradually degraded, which showed that the defect area in the Apt/mGel‐DNA2‐BP100 macroporous hydrogel group was significantly reduced, forming a thin but dense layer of skull tissue.

In the process of bone matrix mineralization, osteocalcin (OCN), as an important marker in the process of bone formation, is mainly secreted by osteoblasts. Immunohistochemistry was used to qualitatively characterize the expression of OCN in the bone defect to study the bone repair performance of the Apt/mGel‐DNA2‐BP100 macroporous hydrogel (Figure S15, Supporting Information). At these three time points, there was no significant difference in the positive expression of OCN in the blank group. At the 2nd and 4th weeks, there was an obvious brownish‐yellow area inside and around the macroporous hydrogel containing BPNSs, that is, the positive expression area of OCN, especially in the Apt/mGel‐DNA2‐BP100 macroporous hydrogel group. These results indicated that Apt and BPNSs synergistically promote the expression of OCN in bone defect areas and the formation of bone mineral matrix.

### 2.7 Molecular Mechanism of Osteogenic Differentiation Regulated by mGel‐DNA‐BP Hydrogel

**Figure 8:**
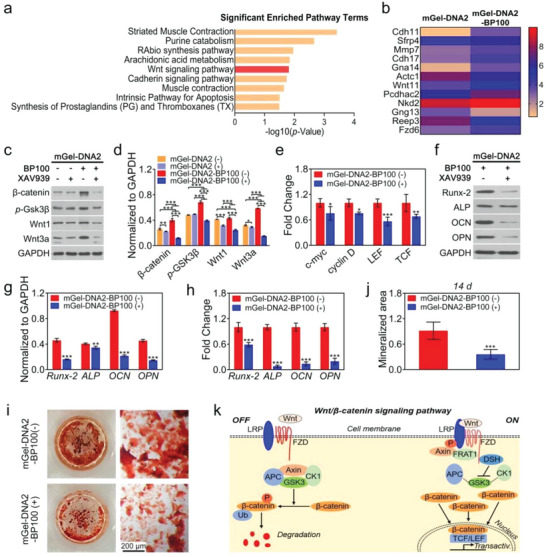
Preliminary analysis of the osteogenic differentiation mechanism. a) Gene chip screening of signaling pathways with significant differences between mGel‐DNA2 and mGel‐DNA2‐BP100 macroporous hydrogels. b) Heatmap of differentially expressed genes in the Wnt signaling pathway in BMSCs. c,d) Expression of Wnt/β‐catenin signaling pathway‐related proteins of BMSCs in mGel‐DNA2 and mGel‐DNA2‐BP100 hydrogels. e) Direct target genes of Wnt/β‐catenin signaling pathway. f–h) Effect of Wnt/β‐catenin signaling pathway inhibitor on genes and proteins expression of BMSCs in mGel‐DNA2‐BP100 hydrogel: (f) Gray value of osteogenic related protein, (g) qualitative analysis of osteogenic related proteins, (h) osteogenesis‐related gene expression, i,j) effect of Wnt/β‐catenin signaling pathway inhibitor on the mineralized matrix of BMSCs cultured on mGel‐DNA2‐BP100 macroporous hydrogel. k) Schematic diagram of the osteogenic differentiation mechanism of BMSCs. ^*^ *p* < 0.05, ^**^ *p* < 0.01, ^***^ *p* < 0.001.

As a complex and dynamic process, bone repair is controlled by many cell signaling pathways.^[^ ^27^ ^]^ We further explored the molecular mechanism by which the mGel‐DNA2‐BP100 macroporous hydrogel induced the osteogenic differentiation of cells. mGel‐DNA2 and mGel‐DNA2‐BP100 macroporous hydrogels were served as the control group and experimental group, respectively. Agilent mRNA expression profiling chip detection was performed on BMSCs (**Figure** **8**a). We found that the Wnt signaling pathway has a key regulatory effect on bone regeneration and bone development, inducing the expression of Wnt signaling pathway‐related genes to be significantly different between mGel‐DNA2 and mGel‐DNA2‐BP100 macroporous hydrogels, as shown in Figure 8b. It was speculated that the mGel‐DNA2‐BP100 macroporous hydrogel may participate in the regulation of the osteogenic differentiation of BMSCs through the Wnt signaling pathway.

XAV939, a small‐molecule inhibitor, can effectively block the Wnt/β‐catenin signaling pathway with concentration‐dependency.^[^ ^28^ ^]^ We selected 2.5 µM XAV939 to block the Wnt/β‐catenin signaling pathway without influencing the viability of BMSCs (Figure S16, Supporting Information). The macroporous hydrogels treated with XAV939 were marked as mGel‐DNA2(+) and mGel‐DNA2‐BP100(+), and the macroporous hydrogels untreated with XAV939 were marked as mGel‐DNA2(−) and mGel‐DNA2‐BP100(−), respectively. A Western blotting assay was performed to detect the expression of the characteristic proteins related to the Wnt/β‐catenin signaling pathway (β‐catenin, *p*‐Gsk3β, Wnt1, and Wnt3a). The expression of these proteins in mGel‐DNA2(−) and mGel‐DNA2‐BP100(−) macroporous hydrogels was significantly higher than that in mGel‐DNA2(+) and mGel‐DNA2‐BP100(+) macroporous hydrogels (Figure 8c,d), indicating that XAV939 successfully blocked the Wnt/β‐catenin pathway. Among them, the gray value of β‐catenin protein in the mGel‐DNA2‐BP100(+) macroporous hydrogel was reduced by 59.5%, inhibiting the activation of β‐catenin on the transcription of downstream Wnt/β‐catenin‐related genes, such as c‐myc, cyclin D, LEF, and TCF (Figure 8e).

Further analysis showed that the osteogenic gene and protein expression of BMSCs cultured with the mGel‐DNA2‐BP100(−) macroporous hydrogel was significantly higher than that of BMSCs cultured with the mGel‐DNA2‐BP100(+) macroporous hydrogel (Figure 8f–h). Specifically, *Runx‐2*, *ALP*, *OCN*, and *OPN* gene expression values of mGel‐DNA2‐BP100(+) decreased to 0.59, 0.08, 0.14, and 0.20 times those of mGel‐DNA2‐BP100(−) macroporous hydrogel, respectively, indicating that blocking the Wnt/β‐catenin signal pathway can inhibit the promotion of mGel‐DNA2‐BP100 macroporous hydrogel on the osteogenic differentiation of cells. Moreover, the blockade of the Wnt/β‐catenin signaling pathway inhibited the promotion of the mGel‐DNA2‐BP100 macroporous hydrogel on the formation of the mineralized matrix of BMSCs (Figure 8i,j), which also revealed that the promoting effect of the mGel‐DNA2‐BP100 macroporous hydrogel on the osteogenic differentiation of BMSCs was related to the activation of the Wnt/β‐catenin signaling pathway. A schematic diagram of the specific mechanism is shown in Figure 8k. The Fzd receptor participated in the interaction of Wnt protein, promoting the accumulation of β‐catenin protein in the cytoplasm to enter the nuclei, and interacting with the transcription factor TCF/LEF to promote the transcription of downstream target genes, such as *Runx‐2*, thereby inducing the osteogenic differentiation of BMSCs.

## 3 Conclusion

In this research, we developed a double‐network macroporous nanocomposite hydrogel with high porosity and mechanical reinforcement by combining the emulsion template method and a double cross‐linking strategy. The distribution of BPNSs in the double‐network macroporous hydrogel can effectively enhance the osteoinductive activity of the matrix, and the biological functionalization of Apt19S based on macroporous hydrogel can accelerate the directional migration of endogenous stem cells to bone defects, thus effectively stimulating their osteogenic differentiation and accelerating bone regeneration and repair. We believe that our research results provide strong evidence that an effective method has been developed to use BPNSs as a phosphorus‐based preparation to achieve the improvement of the osteoinductive activity of the hydrogel, and the combination of biomolecules, nanomaterials, and the field of tissue regeneration through the design of macroporous hydrogels is expected to provide new construction strategies for future biomaterials for bone repair.

## 4 Experimental Section

### Preparation of Double‐Network Macroporous Hydrogel

Briefly, DNA (deoxyribonucleic acid sodium salt from salmon testes, molecular weight ≈1.3 × 10^6^ g mol^−1^, corresponding to ≈2000 base pairs, Sigma–Aldrich, USA) was added to the aqueous GelMA solution (10 w/v%) containing 0.5 w/v% hydroxy‐4′‐(2‐hydroxyethoxy)−2‐methyl‐propiophenone (I2959, Sigma–Aldrich, USA) and stirred at 40 °C until completely dissolved to obtain composite pre‐gels. GelMA with 80% of methacrylation was synthesized as described previously.^[^ ^29^ ^]^ 200 µL of the obtained pre‐gels were treated at 95 °C for 5 min and then emulsified by a homogenizer (IKA T10 Basic) to prepare air‐in‐water emulsions. The emulsions were further polymerized by UV irradiation (365 nm, 60 s) to produce the macroporous DNA composite hydrogels, where the final DNA content was 0, 1.0, 2.0, and 4.0 w/v% and termed as mGel, mGel‐DNA1, mGel‐DNA2, and mGel‐DNA4, respectively.

### Characterization of the Double‐Network Macroporous Hydrogel

The hydrogels were lyophilized and sputter‐coated with gold, and the internal morphology of the macroporous hydrogels was observed by scanning electron microscopy (SEM, Leica, Merlin, Germany). Micro‐CT system (Metris, HARRIER HP23.4JX, UK) was also used to characterize the porous structure in 3D to analyze the porosity and pore size distribution.

Rheometer (MCR 302, Anton Paar) was used to evaluate the mechanical properties of the hydrogels. Strain sweep testing was conducted to analyze the storage modulus (*G*’) and loss modulus (*G*″) of hydrogels in the range of 0.01–2000% shearing strain. Oscillation frequency sweep testing was carried out to determine the corresponding *G*’ and *G*″ in the range of 0.01–100 rad s^−1^. The macroporous hydrogels were prepared into a cylindrical shape with a diameter of ≈10 mm and a thickness of ≈5 mm. Dynamic mechanical testing (DMA Q800, TA Instruments, USA) was also applied to evaluate the mechanical properties of the hydrogels. In the static compression mode, the compression test was performed at a strain rate of 10% min^−1^. For the dynamic compression test, the strain was increased from 0% to 60% and then restored to 0 to complete a cycle, and 5 cycle tests were conducted.

To determine the in vitro degradation of the macroporous hydrogels, purified hydrogels with different DNA concentrations were lyophilized and the dry weight was recorded (*W*_d_). The dried hydrogel was soaked in collagenase solution (1 µg mL^−1^). At the predetermined time, the degraded hydrogels were collected. The samples were freeze‐dried and weighed (*W*_r_). The mass loss ratio was calculated by: (W_d_ − W_r_) / W_d_ × 100%.

The original mass of freeze‐dried hydrogels was recorded as *M*_o_. After that, the hydrogels were immersed in PBS at room temperature. At a predetermined time, the hydrogels were removed from the PBS and freeze‐dried, then weighed and recorded as *M*_t_. The swelling ratio (*Q*) of the hydrogels was calculated by the following equation: *Q* = (*M*_t_ − *M*_o_)/*M*_o_.

### Construction of mGel‐DNA‐BPNSs Macroporous Hydrogel

The preparation procedure of BPNSs‐loaded hydrogels was similar to that of the mGel‐DNA composite hydrogel. The BPNSs contents in the hydrogels were 0, 50, 100, and 200 ppm, and termed as mGel‐DNA, mGel‐DNA‐BP50, mGel‐DNA‐BP100, and mGel‐DNA‐BP200, respectively.

### Cell Viability and Proliferation Assessment

Rat bone marrow mesenchymal stem cells (BMSCs) were purchased from the Type Culture Collection of the Chinese Academy of Sciences (Shanghai, China). BMSCs were cultured in Dulbecco's modified Eagle medium (DMEM, Gibco, USA) containing 10% fetal bovine serum (Gibco, USA) and 1% penicillin‐streptomycin (Gibco, USA) at 37 °C under 5% CO_2_. BMSCs were trypsinized and collected by centrifugation for passage and seeding.

The prepared hydrogel samples were placed in a 48‐well plate, and the effect of mGel‐DNA‐BP macroporous hydrogels on cell proliferation was evaluated by the Cell Counting Kit‐8 (CCK‐8) proliferation experiment. BMSCs were seeded on the hydrogels in a 48‐well plate at a density of 5 × 10^4^ cells/well and cultured in an incubator (37 °C, 5 v/v% CO_2_) for 1, 3, and 5 days, respectively. At a predetermined time, the samples were transferred to a new 48‐well plate. The medium and CCK‐8 stock solution (Dojindo Laboratories, Japan) were prepared into working solution at a volume ratio of 10:1, and 200 µL of CCK‐8 working solution was added to each well and placed in an incubator for 90 min. Then, 110 µL of incubation solution was pipetted into a 96‐well plate, and the OD values were measured at a wavelength of 450 nm with a microplate reader (Thermo 3001, Thermo Scientific, USA). In addition, live‐dead staining was also used to characterize the cell viability of BMSCs on the hydrogels. Calcein‐AM staining solution and PI staining solution were diluted in PBS at a volume ratio of 1.5:1000 and 0.5:1000, respectively, and 500 µL mixed staining solution was added to the wells containing samples and incubated for 30 min at room temperature in the dark. After staining, PBS was used to remove the remaining staining solution on the samples, and the samples were observed by confocal laser scanning microscopy (CLSM, Leica TCS SP8, Germany).

### Cell Infiltration

To study the distribution of cells in the macroporous hydrogel, BMSCs were seeded on *N*‐mGel‐DNA nonporous hydrogel, mGel‐DNA, and mGel‐DNA‐BP macroporous hydrogels in a 24‐well plate at an amount of 1 × 10^5^ cells per well, and the medium was changed every other day. After culturing for 3 days, calcein‐AM staining solution was diluted in PBS at a volume ratio of 1.5:1000, and was added to the wells containing samples to incubate for 30 min in the dark at room temperature. After the incubation, PBS was used to remove the residual staining solution, and the fluorescence images were captured by CLSM under the excitation wavelength of 488 nm.

### In Vitro Osteogenic Differentiation of Cells

BMSCs (1 × 10^5^ cells per well) were seeded on *N*‐mGel‐DNA nonporous hydrogel, mGel‐DNA, and *N*‐mGel‐DNA‐BP macroporous hydrogels in a 24‐well plate. After 7 days and 14 days of culture, the expression of osteogenic genes (*Runx‐2*, *ALP*, *OCN*, and *OPN*) was detected by quantitative real‐time polymerase chain reaction (qRT‐PCR). The primer sequences are shown in Table S2 (Supporting Information). After 14 days of culture, the formation of mineralized matrix was characterized by Alizarin Red staining. Before staining, BMSCs on the hydrogels were trypsinized and collected by centrifugation. Then, the mineralized matrix of BMSCs was labeled with Alizarin Red solution, and the mineralized matrix was observed using an optical microscope. After that, 100 mm dodecylpyridinium chloride solution was added to each well to quantify the amount of mineralized matrix. Finally, a microplate reader (Thermo 3001, Thermo Scientific, USA) was used to measure the absorbance at 562 nm.

### Molecular Mechanism Analysis

To clarify the regulatory mechanism of the mGel‐DNA‐BP macroporous hydrogel on the osteogenic differentiation of BMSCs, Agilent mRNA expression profiling chip was used to determine the signaling pathway and expression level of key genes. The brief steps were as follows: RNA from BMSCs was extracted using Trizol reagent (Life technologies, USA), and then amplified and labeled in vitro. The hybridization images were obtained using an Agilent chip scanner and the results were analyzed using agilent feature extraction software. XAV939 was a small‐molecule inhibitor that could selectively inhibit the Wnt/β‐catenin signaling pathway.^[^ ^28^ ^]^ XAV939 (S1180, Selleck, USA) was dissolved in DMSO to prepare stock solution. Then, 20 µL of XAV939 (5 mm) was added to 40 mL DMEM containing 10% fetal bovine serum and 1% penicillin‐streptomycin to obtain XAV939 (2.5 µm) medium. In addition, 40 mL DMEM containing 10% fetal bovine serum and 1% penicillin‐streptomycin was collected, and 20 µL DMSO was added to prepare a blank medium. BMSCs were seeded on the mGel‐DNA and mGel‐DNA‐BP hydrogels in a 24‐well plate at an amount of 1 × 10^5^ cells per well and placed in a 37 °C incubator. After 24 h of culture, blank medium and XAV939 medium were supplemented, qRT‐PCR and western blotting analysis were performed after 7 days of cell culturing, and Alizarin red staining was performed after 14 days of cell culturing. The primer sequences are shown in Tables S2 and S3 (Supporting Information).

### Apt19S Combined with BPNSs in Macroporous Hydrogels Regulating Bone Regeneration

Apt19S‐functionalized mGel‐DNA‐BP macroporous hydrogels. DNA was added to GelMA aqueous solution (15 wt.%) containing 0.5 w/v% I2959 and stirred at 40 °C until completely dissolved to obtain the pre‐gel. Pre‐gel (200 µL) was treated at 95 °C for 5 min, and 100 µL of an aqueous mixture of Apt19S and BPNSs was added. The final concentrations of Apt19S and BPNSs in the pre‐gel were 5 µm and 100 ppm, respectively. The Apt19S sequence is shown in Table S4 (Supporting Information). The pre‐gel was emulsified and further polymerized by UV irradiation to produce Apt/mGel‐DNA‐BP macroporous hydrogel.

### In Vitro Cell Chemotaxis

A transwell experiment was conducted to characterize the chemotactic effect of macroporous hydrogels on BMSCs. First, macroporous hydrogel was placed in the lower chamber, 200 µL of 1 × 10^5^ cells mL^−1^ serum‐free cell suspension was added to the upper chamber, and 600 µL DMEM containing 10% FBS was added to the lower chamber. After 36 h, BMSCs were fixed with 4% paraformaldehyde for 30 min. Then, 0.1% crystal violet solution was added into the lower chamber to label BMSCs for 20 min. After that, PBS was used to wash the samples, cotton swabs were used to remove BMSCs on the upper surface of the membrane, and then the BMSCs on the lower surface of the membrane were observed using an optical microscope. Five different fields of view were captured in each group, and the number of BMSCs passing through the lower surface of the membrane was analyzed by Image J program.

### In Vivo Cell Recruitment

The BMSCs recruitment of the Apt/mGel‐DNA‐BP macroporous hydrogel was evaluated by the Sprague Dawley rat (6 weeks, female) cranial defect model. The animal experimental procedures were performed in accordance with the Guidelines for care and use of Laboratory Animals of Guangdong Academy of Medical Sciences and approved by the Ethics Committee for Animal Experiment of Guangdong Academy of Medical Sciences (No. KY‐Z‐2022‐380‐01). Cranial defects with a diameter of 5 mm were created on both sides of each rat's cranium, with four rats per group at each time point. mGel‐DNA, mGel‐DNA‐BP, and Apt/mGel‐DNA‐BP macroporous hydrogels were implanted into the cranial defect, respectively, and the cranial defect without implanted material was regarded as the blank group. On day 7, the cell antigens (CD29, CD44, CD45) on the implanted material were analyzed by flow cytometry, and the percentage of BMSCs was calculated. The material removed from the vivo was transferred to 1.5 mL centrifuge tube. After that, the cells were digested with trypsin, and 1 mL PBS was added to wash the cells by centrifugation. The cell suspension was divided equally for fluorescent labeling and unlabeled control groups. The cell suspension to be fluorescently labeled was incubated with monoclonal anti‐CD29, anti‐CD44, and anti‐CD45 antibodies for 30 min on ice. After incubation, 500 µL of cell suspension was taken for flow cytometric analysis. The unlabeled control group was regarded as the negative screening standard. CD29 and CD44 double‐positive cells were first selected, and then CD45‐negative cells from these double‐positive cells were identified to calculate the percentage of the selected cells to the total number of cells, which was regarded as the proportion of BMSCs. Meanwhile, immunofluorescence staining was used to characterize the expression of CD29 and CD44 in the cranial defect, and the digital pathology scanning system was used to collect images for observation.

### In Vivo Bone Regeneration

Sprague Dawley rats were sacrificed at the 2nd, 4th, and 8th weeks, and the craniums were taken out for micro‐CT system analysis. The ZKKS‐MicroCT4.1 program was used to calculate the relevant bone parameters, including new bone volume (*BV*), new bone volume/defect volume (*BV*/*TV*), and new bone density (*BMD*). To observe the cellular and tissue response to various treatments, the cranium tissue was embedded in paraffin to prepare tissue sections, and then hematoxylin and eosin (H&E) staining and Masson trichrome staining were performed.

### Statistical Analysis

Data requiring statistical analyses were evaluated using the Prism 6 program (GraphPad Software, La Jolla, USA). All the data obtained were expressed as means ± standard deviation (*SD*) and were analyzed using one‐way ANOVA with a post hoc test. Differences between the experimental and control groups were compared. Groups with *p* < 0.05 were marked as significantly different (^*^ *p* < 0.05, ^**^ *p* < 0.01, ^***^ *p* < 0.001).

## Conflict of Interest

The authors declare no conflict of interest.

## Supporting information

Supporting Information

## Data Availability

The data that support the findings of this study are available from the corresponding author upon reasonable request.
