# Supplementary material for: Double‐Network DNA Macroporous Hydrogel Enables Aptamer‐Directed Cell Recruitment to Accelerate Bone Healing
Source: Adv Sci (Weinh). 2023 Nov 10;11(1):2303637. doi: 10.1002/advs.202303637 (PMC10767401; doi:10.1002/advs.202303637)
Supplement: Supplementary file 1 — Supporting Information [file ADVS-11-2303637-s001.pdf]

ADVANCED SCIENCE
Open Access

Supporting Information

for *Adv. Sci.*, DOI 10.1002/advs.202303637

Double-Network DNA Macroporous Hydrogel Enables Aptamer-Directed Cell Recruitment to Accelerate Bone Healing

Yali Miao, Xiao Liu, Jinshui Luo, Qian Yang, Yunhua Chen and Yingjun Wang**

Supporting Information

Double-Network DNA Macroporous Hydrogel Enables Aptamer-Directed Cell Recruitment to Accelerate Bone Healing

Yali Miao, Xiao Liu, Jinshui Luo, Qian Yang, Yunhua Chen, and Yingjun Wang**

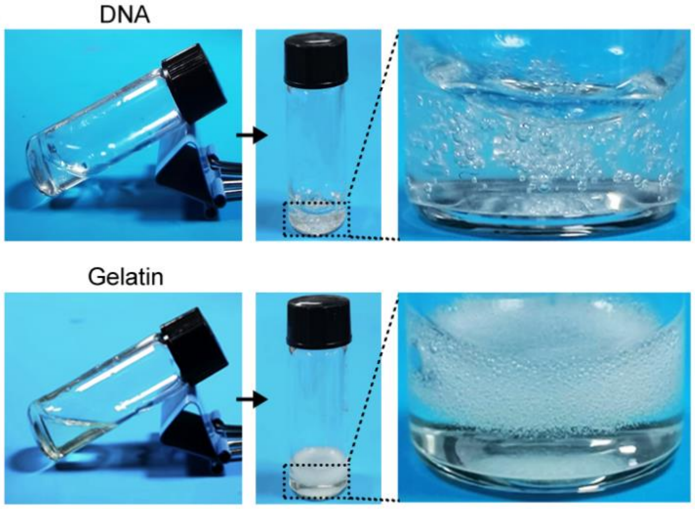

Figure S1. DNA and gelatin solutions before and after emulsification, indicating that the individual DNA and gelatin solution cannot form emulsion form through high-speed shearing.

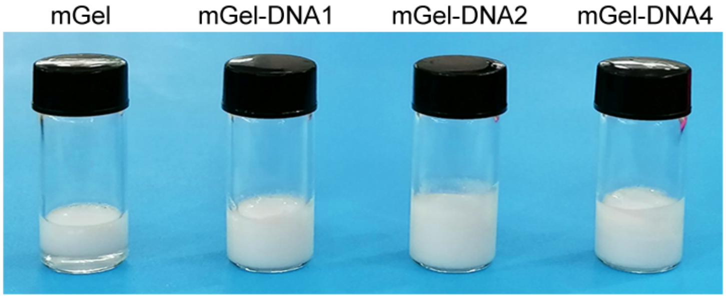

Figure S2. Photographs of mGel and mGel-DNA emulsions after standing for 12 hours.

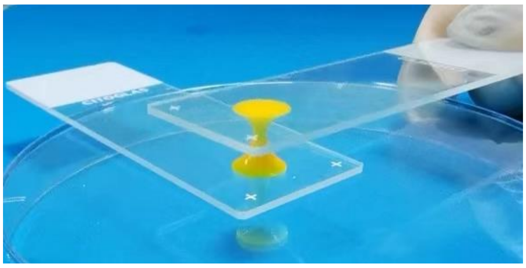

Figure S3. mGel-DNA2 emulsion droplets (stained with tartrazine) contacted each other with obvious interaction effect.

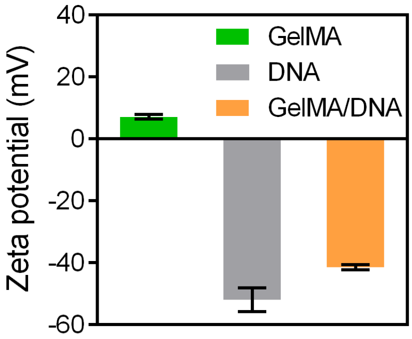

Figure S4. Zeta potential of DNA, GelMA and the mixture at pH 7.0.

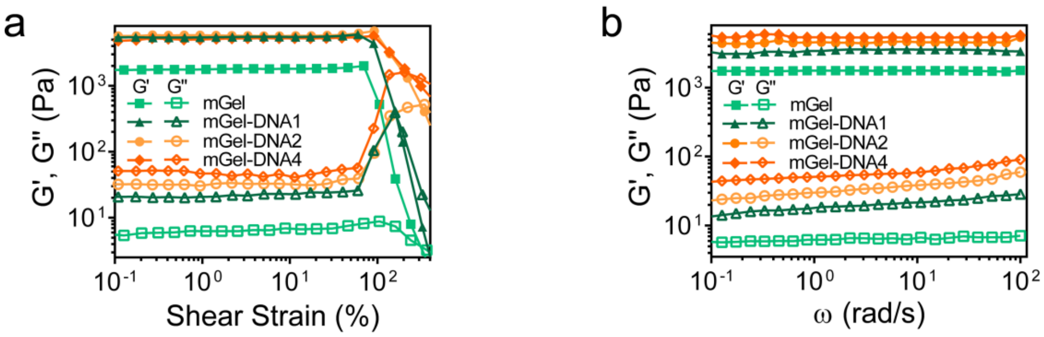

Figure S5. (a) Strain sweep curves and (b) oscillation frequency sweep curves of mGel and mGel-DNA hydrogels.

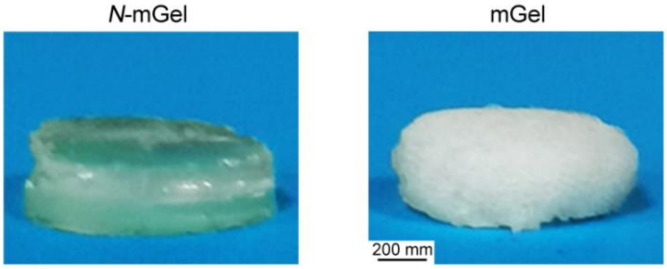

Figure S6. Photographs of *N*-mGel nonporous hydrogels and mGel macroporous hydrogels.

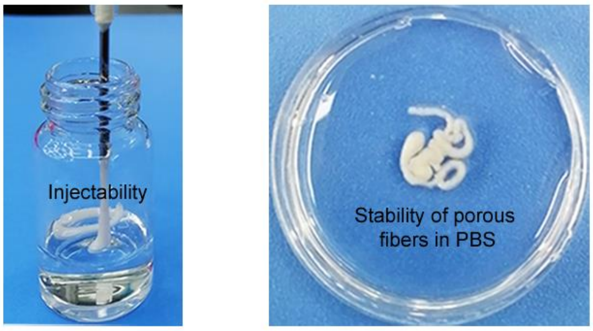

Figure S7. Injectability and stability of mGel-DNA2 emulsion.

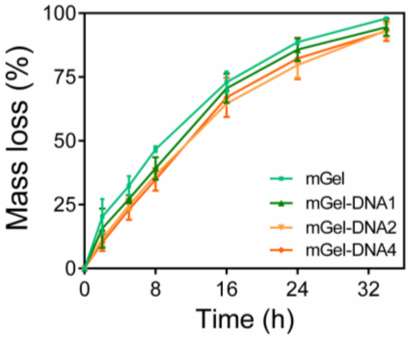

Figure S8. Degradation profiles of mGel-DNA macroporous hydrogels, *$p < 0.05$, **$p < 0.01$, ***$p < 0.001$.

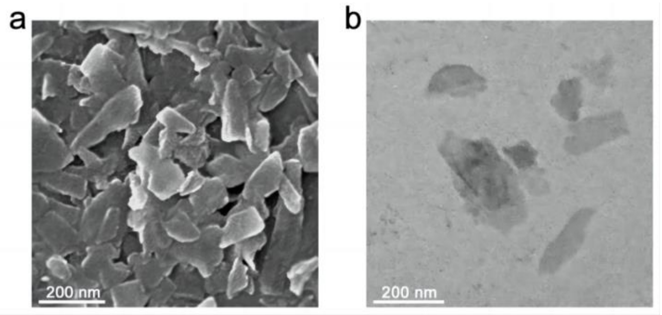

Figure S9. Morphological characterization of BPNSs. (a) SEM image and (b) TEM image of BPNSs.

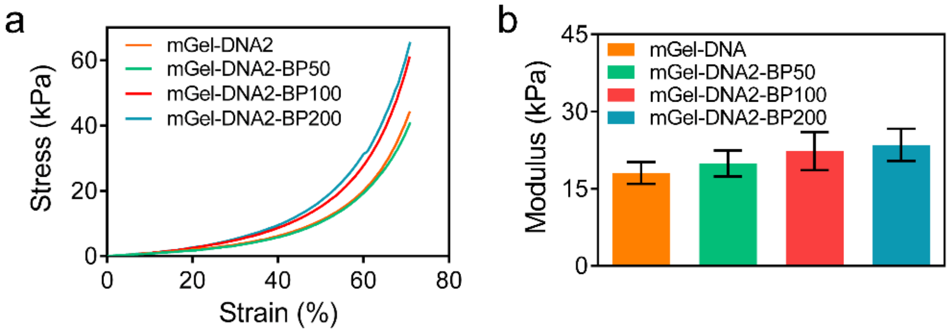

Figure S10. Effect of BPNSs on the mechanical properties of mGel-DNA2 macroporous hydrogel. (a) stress-strain curves, (b) compressive modulus.

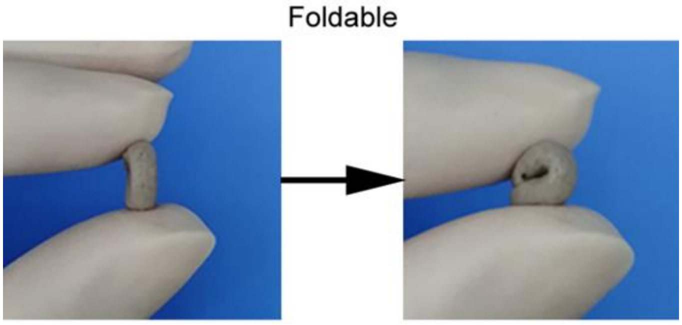

Figure S11. Photographs of mGel-DNA2-BP100 macroporous hydrogel before and after folding.

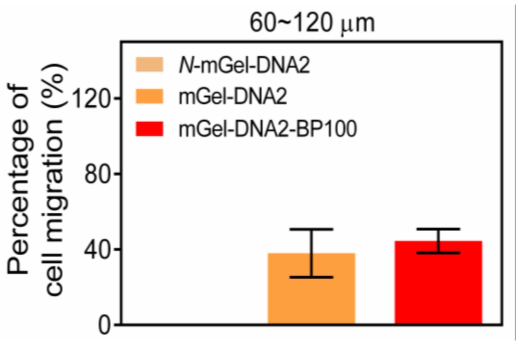

Figure S12. Distribution percentage of BMSCs in the depth range of 60~120 μm, *$p < 0.05$, **$p < 0.01$, ***$p < 0.001$.

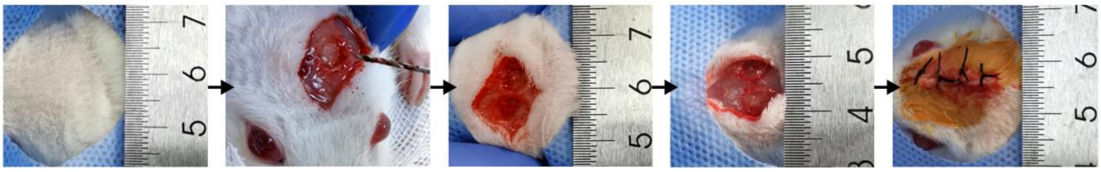

Figure S13. Construction process of cranial defect in rats.

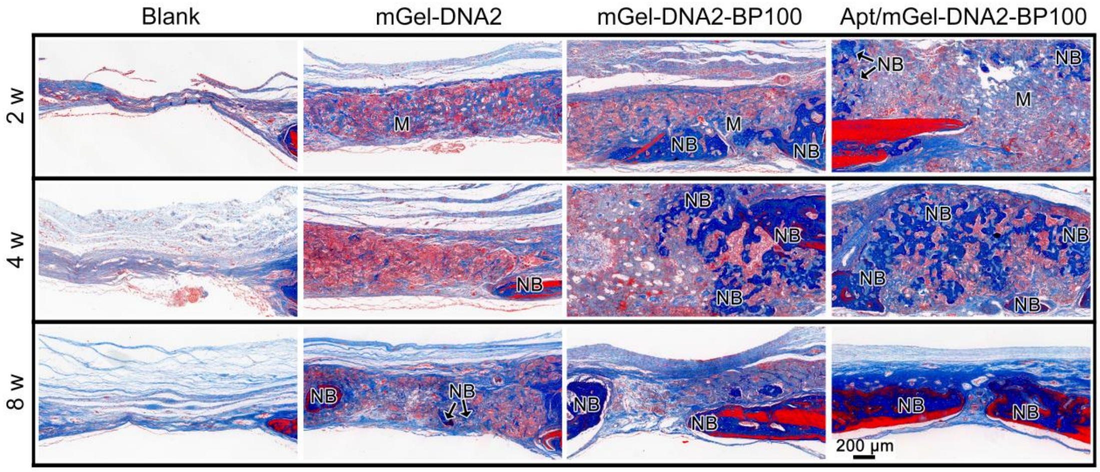

Figure S14. Masson staining images of regenerated bone tissue, NB stands for new bone, M stands for implant materials.

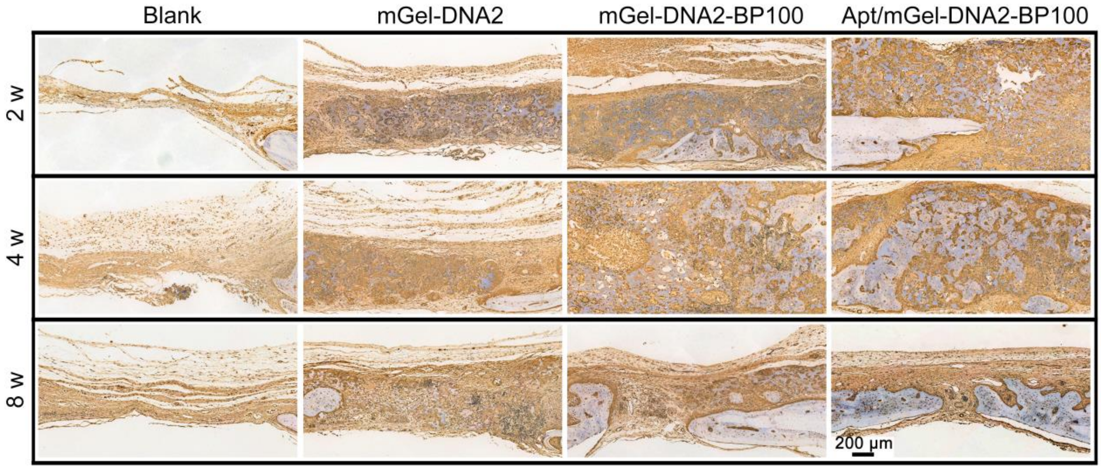

Figure S15. OCN immunohistochemical staining images of regenerated bone tissue at the cranial defects.

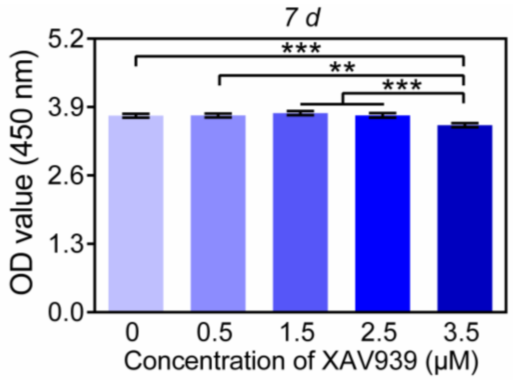

Figure S16. Effect of different concentrations of XAV939 on viability of BMSCs, $*p < 0.05$, $**p < 0.01$, $***p < 0.001$.

Table S1. The specific content of BPNSs in mGel-DNA2 hydrogels

**Sample**	**BPNSs concentration (ppm)**
N-mGel-DNA2	0
mGel-DNA2	0
mGel-DNA2-BP50	50
mGel-DNA2-BP100	100
mGel-DNA2-BP200	200

Table S2 Oligonucleotide primer sequences utilized for qRT-PCR

**Target DNA**	**Primer sequence (5'-3')**
GAPDH	F: CACCCAGCCCAGCAAGGATA<br>R: GCCCCTCCTGTTGTTATGG
Runx-2	F: TCCTTCCCTCCGAGACCCTA<br>R: GGTCAGTCAGTGCCTTTCCTT
ALP	F: GCCAGGCTGGGAAGAACAAC<br>R: ACAGGGGAGTCGCTTCAGTG
OPN	F: GGCCCTGAGCTTAGTTCGTTG<br>R: GCAGTGGCCATTTGCATTTC
OCN	F: GGCGCTACCTCAACAATGGAC<br>R: CGTCCTGGAAGCCAATGTG
VEGF	F: CCTGCAGCATAGCAGATGTGAAT<br>R: GTCTAGTTCCCGAAACCCTGAG

Table S3 Oligonucleotide primer sequences utilized for qRT-PCR

**Target DNA**	**Primer sequence (5'-3')**
GAPDH	F: TTGGCTACAGCAACAGGGTG<br>R: TCTTCCTCTTGTGCTCTTGCTG
C-myc	F: TACTGAGGAAACGGCGAGAA<br>R: AACCGTTCTCCTTACACTCG
Cyclin D	F: TGCTGGCGAAGGTTTAGG<br>R: GTTGGGAAAGTCAAGGAGA
LEF	F: GGTCAGCCTGTTTATCCC<br>R: TGAGGGATGCCAGTTGTG
TCF	F: CACTGAGAAGCCGCAGACCA<br>R: TGCTTTGGGGTTCAGGTT

Table S4. Apt19S sequence

**Target ssDNA**	**Primer sequence (5'-3')**
5'-acrydite-Apt19S	5'-acrydite-(A)9-AGGTCAGATGAGGAGGGGGA<br>CTTAGGACTGGGTTTATGACCTATGCGTG-3'
